# Decoding Task-Related Functional Brain Imaging Data to Identify Developmental Disorders: The Case of Congenital Amusia

**DOI:** 10.3389/fnins.2019.01165

**Published:** 2019-10-30

**Authors:** Philippe Albouy, Anne Caclin, Sam V. Norman-Haignere, Yohana Lévêque, Isabelle Peretz, Barbara Tillmann, Robert J. Zatorre

**Affiliations:** ^1^Cognitive Neuroscience Unit, Montreal Neurological Institute, McGill University, Montreal, QC, Canada; ^2^International Laboratory for Brain, Music and Sound Research, Montreal, QC, Canada; ^3^INSERM, U1028, CNRS, UMR 5292, Lyon Neuroscience Research Center, Brain Dynamics and Cognition Team, Lyon, France; ^4^University Lyon 1, Lyon, France; ^5^Zuckerman Institute of Mind, Brain and Behavior, Columbia University, New York, NY, United States; ^6^CNRS, Laboratoire des Sytèmes Perceptifs, Département d’Études Cognitives, ENS, PSL University, Paris, France; ^7^CNRS, UMR 5292, INSERM, U1028, Lyon Neuroscience Research Center, Auditory Cognition and Psychoacoustics Team, Lyon, France

**Keywords:** multivariate pattern analysis (MVPA), rs-fMRI, sMRI, task-based fMRI, tone deafness, diagnostic, brain-based biomarkers

## Abstract

Machine learning classification techniques are frequently applied to structural and resting-state fMRI data to identify brain-based biomarkers for developmental disorders. However, task-related fMRI has rarely been used as a diagnostic tool. Here, we used structural MRI, resting-state connectivity and task-based fMRI data to detect congenital amusia, a pitch-specific developmental disorder. All approaches discriminated amusics from controls in meaningful brain networks at similar levels of accuracy. Interestingly, the classifier outcome was specific to deficit-related neural circuits, as the group classification failed for fMRI data acquired during a verbal task for which amusics were unimpaired. Most importantly, classifier outputs of task-related fMRI data predicted individual behavioral performance on an independent pitch-based task, while this relationship was not observed for structural or resting-state data. These results suggest that task-related imaging data can potentially be used as a powerful diagnostic tool to identify developmental disorders as they allow for the prediction of symptom severity.

## Introduction

One of the main challenges of brain imaging is to provide individual discrimination ability to inform diagnosis and prognosis of neurodegenerative or developmental disorders at the individual level (Uddin et al., 2017). A growing number of studies have used machine learning classification techniques on either structural MRI (sMRI) or resting-state fMRI (rs-fMRI) data to identify brain-based disorder-related biomarkers (Arbabshirani et al., 2017; Uddin et al., 2017). These methods have shown great potential in discriminating abnormal development, such as Autism Spectrum Disorder, attention-deficit hyperactivity disorder or dyslexia, from typical development (Bray et al., 2009; Arbabshirani et al., 2017). While some studies have shown that decoding performed on task-related fMRI can yield similar accuracy to sMRI and rs-fMRI in classifying clinical populations (Shenas et al., 2014; Bruin et al., 2018), the power of task-related fMRI as a diagnostic approach has been, to date, somewhat neglected. This is mainly related to the fact that, unlike task-based fMRI, sMRI, and rs-fMRI imaging data can be easily acquired from otherwise difficult-to-scan populations in a relatively short recording period of time (Bruin et al., 2018). Here, in contrast, we hypothesized that task-based fMRI may present significant advantages in relating classifier outcomes to phenotypic or behavioral measures as compared to sMRI and rs-fMRI data because of the potential specificity they offer to probe brain activity.

In the present study, we used all three approaches (sMRI, rs-fMRI, and task-fMRI) to perform imaging-based classification of congenital amusia, a developmental disorder of the central auditory system resulting in behavioral impairments of pitch perception and memory (Albouy et al., 2013a, 2019; Peretz, 2016; Tillmann et al., 2016). These behavioral deficits have been linked to anatomical abnormalities along the right fronto-temporal pathway, notably in terms of white and gray matter concentration in the right inferior frontal gyrus (Hyde et al., 2006; Albouy et al., 2013a), and in the right superior temporal gyrus (Hyde et al., 2007; Albouy et al., 2013a), as well as the structural connectivity between these regions (Loui et al., 2009). Functional investigations have reported abnormal responses of the right fronto-temporal pathway including the auditory cortex and the IFG during pitch perception (Hyde et al., 2011) and pitch memory (Albouy et al., 2013a, 2015, 2019; Tillmann et al., 2016). During resting state, abnormally increased connectivity between the auditory cortices and the Default Mode Network (DMN, a network of areas showing greater activation during rest than during goal-directed tasks (Raichle et al., 2001; Greicius et al., 2003) has been reported in congenital amusia (Leveque et al., 2016). Thus, activity in the fronto-temporal network during task performance, and in auditory and DMN networks during resting state might serve as indexes of the degree of impairment in an individual. In contrast, amusics show normal memory performance for mono-syllabic words spoken with a constant pitch (Tillmann et al., 2009; Albouy et al., 2019) and spoken numbers (digits spans, Williamson and Stewart, 2010; Albouy et al., 2013b) as well as intact (i.e., similar to controls) left fronto-temporal network activation during verbal memory (Caclin and Tillmann, 2018; Albouy et al., 2019).

In the present study, we investigated whether amusic individuals can be discriminated from control participants using whole-brain multivariate pattern analysis applied on sMRI, resting-state functional connectivity, and task-related fMRI. Based on previous studies reported above, we hypothesized that the classifier will be able to discriminate amusics and controls with sMRI, rs-fMRI and pitch-based task-related fMRI data. In contrast, we expected the classifier to fail in discriminating amusics and controls for the task-fMRI data acquired during verbal memory (sequences of mono-syllabic words spoken with a fixed pitch of 230 Hz). Finally, by extracting classifier decision values (distance from the separating hyperplane) and by relating them to a behavioral score acquired independently, we tested if we could predict the severity of behavioral deficits in individual participants. We hypothesized that pitch-based task-fMRI, which captures brain dynamics that are specifically related to the behavioral correlates, may present significant advantages, in relating classifier outcomes to behavioral measures.

## Materials and Methods

### Participants

Eighteen amusic adults and 18 non-musician controls matched for gender, age, handedness, years of education, and years of musical instruction, participated in the study (see details in Table 1). The amusic group was composed of 13 participants from Lyon (France) and five from Montreal (Canada). The control group was composed of 14 participants from Lyon and four from Montreal. All participants had right-handed laterality and reported no history of dyslexia, nor history of neurological or psychiatric disease. They gave their written informed consent and received a monetary compensation for their participation. All participants were tested with standard audiometry and none of them had moderate (35 dB) or severe (more than 40 dB) peripheral hearing loss at the frequencies of interest (between 250 to 1000 Hz). All participants had been thoroughly evaluated on previous testing sessions with the Montreal Battery of Evaluation of Amusia (MBEA see Table 1, Peretz et al., 2003). Participants were considered amusic when they scored below 23 across the six tasks of the battery (maximum score = 30), the cut-off being two standard deviations below the average of the normal population (see Table 1).

**TABLE 1:** Demographic characteristics of the full sample of amusics and controls.

**Demographic**	**Amusics**	**Controls**	***t*-Test**
**characteristics**	**(*n* = 18)**	**(*n* = 18)**	
Age in years	42.4 (14.6)	40.8 (14.0)	*p* = 0.74 (NS)
Gender	11F, 7M	11F, 7M	N/A
Education in years	15.0 (3.6)	13.9 (3.1)	*p* = 0.33 (NS)
Musical education in years	0.83 (1.4)	0.33 (1.0)	*p* = 0.23 (NS)
MBEA (Peretz et al., 2003)	20.9 (1.5)	26.7 (1.4)	*p* < 0.0001
PDT (Tillmann et al., 2009)	0.90 (0.88)	0.22 (0.15)	*p* = 0.002

*Results on the Montreal Battery of Evaluation of Amusia (MBEA) are expressed as number of correct responses (average over the six sub-tests of the battery, maximum score = 30). Pitch Discrimination Thresholds (PDT) are in semitones. Data are reported as a function of group and groups are compared with *t*-tests. “NS” refers to a non-significant difference (*p* > 0.05) and standard deviations are in parentheses.*

To evaluate pitch discrimination thresholds (PDTs), all participants were tested with a two-alternative forced-choice task using a two-down/one-up adaptive staircase procedure (see Tillmann et al., 2009 for details). The average PDT of the amusic group (ranging from 0.13 to 2.41 semitones) was significantly higher [worse, *t*(34) = 3.23, *p* = 0.002] than that of the control group (ranging from 0.05 to 0.67 semitones). In agreement with previous findings (Tillmann et al., 2009, 2016), we observed a partial overlap in PDTs between amusic and control groups.

These 36 subjects have participated to the structural imaging (T1-MPRAGE) and Task-fMRI for short-term memory (tonal and verbal tasks). Note that only a subset of subjects has participated to the rs-fMRI (13 in each group, all participants were recorded in Lyon) and task fMRI: pitch localizer (*n* = 12 in each group, all participants were recorded in Lyon). All data (in both Montreal and Lyon) were collected using a 3T Philips Achieva TX scanner with a 32-channel head coil. The study was approved by French and Canadian local ethics committees on Human Research and all participants gave written informed consent.

### Structural MRI

#### Participants

The full sample (36 subjects) has participated in this protocol. See Table 1 for details.

#### Image Preprocessing and Segmentation

High-resolution T1-weighted three-dimensional (3D) images were acquired using a gradient-echo sequence [160 sagittal slices; time to repetition (TR) = 2800 ms; time to echo (TE) = 3.8 ms; flip angle = 8°; matrix size = 240 × 240; field of view = 240 mm × 240 mm; voxel size = 1 mm × 1 mm × 1 mm]. All image preprocessing were performed using the VBM functions of SPM12 (Wellcome Trust Centre for Neuroimaging^1^, London, United Kingdom). Before preprocessing, all images were checked for artifacts and automatically aligned so that the origin of the coordinate system was located at the anterior commissure. Using the unified segmentation procedure implemented in SPM12 (Ashburner and Friston, 2005), the images were segmented into gray matter, white matter, and cerebrospinal fluid. For each participant, this resulted in a set of three images in the same space as the original T1-weighted image, in which each voxel was assigned a probability of being gray matter, white matter, and cerebrospinal fluid, respectively, as well as a normalized version of these images (using the T1-template from the Montreal Neurological Institute, provided by SPM12).

### Resting State fMRI

#### Participants

Only a subset of subjects has participated to the rs-fMRI (13 in each group), all participants were recorded in Lyon. See Supplementary Table S1 for details.

#### Data Acquisition and Procedure

Twelve minutes of functional resting-state scans were acquired using an interleaved 2D T2^∗^ SENSE echo planar imaging (EPI) sequence with the sequence parameters of Fauvel et al. (2014): 2D T2^∗^-FFE-EPI axial, SENSE factor = 2, TR = 2,382 ms, TE = 30 ms, flip angle = 80°, 42 slices, slice thickness = 2.8 mm, no gap, in-plane resolution = 2.8 mm × 2.8 mm, 280 volumes. During the resting-state acquisition, participants were required to keep their eyes closed and stay awake. In the debriefing interview after the scanning session, all participants reported they were indeed able to stay awake.

#### Data Analysis

Except for seed determination (see below), we used an adaptation of the processing pipeline of Fauvel et al. (2014) with SPM8^1^ (Wellcome Department of Imaging Neuroscience Group, London, United Kingdom). Each functional volume series was automatically inspected for excessive head movements with the tsdiffana routine^2^. No abnormal spike of variance, rotational (>1.5°) or translational (>3 mm) movement, was observed in time series in each group. T1-weighted structural images were spatially normalized to the Montreal Neurological Institute (MNI) template (ICBM AVG152), segmented using VBM8^3^, and smoothed using an 8-mm full width at half maximum (FWHM) isotropic Gaussian kernel. EPI volumes were corrected for slice timing, realigned on the first volume, and coregistered to the T1 volume (see Structural MRI). The coregistered T1 and EPI volumes were normalized on the basis of the segmented gray matter, and 4-mm FWHM smoothing was applied to the EPI volumes. The signal was bandpass filtered (0.01–0.08 Hz). Finally, the individual segmented gray matter T1 volumes were averaged in the MNI space, and a binary mask was created including only voxels with values above 0.3 in the average gray matter image and with a higher probability to be gray matter than white matter or cerebrospinal fluid. This binary mask was used in all subsequent analyses.

#### Seed Determination

We used functionally defined seeds that were 10-mm-diameter spheres in right and left Heschl’s gyri, centered on the MNI coordinates (x = 45 y = −19 z = 6) and (x = −44 y = −18 z = 5) observed in the magnetoencephalographic (MEG) data of Albouy et al. (2013a). The seeds correspond to the sources of the N100 responses for tone encoding, where significant differences of activity and of connectivity with frontal areas were observed between amusics and controls. The entire sphere was located within the most medial part of Heschl’s gyri of each individual’s anatomical MRI and did not overlap with other non-auditory regions.

#### Resting-State Analysis

For each participant, the time series were extracted and averaged across voxels within the seed regions with the MarsBaR toolbox (Brett et al., 2002), and the correlations between the seed time series and the time series of all other voxels of the entire brain gray matter mask were calculated, with motion parameters, white matter (WM), and cerebrospinal fluid (CSF) time series as regressors of non-interest. To extract the WM and CSF time series, WM and CSF masks were computed by thresholding the mean of the spatially normalized WM and CSF images (≥1) with ImCalc (SPM8). These masks were then eroded by three voxels along each of the three axes with Anatomist^4^. Individual connectivity maps were then transformed into Z-score maps, with connectivity defined as a pairwise correlation between the seed time-series and the time-series of other voxels.

### Task-fMRI: Short-Term Memory

#### Participants

The full sample (36 subjects) has participated in this protocol. See Table 1 for details.

#### Stimuli

**FIGURE 1:**
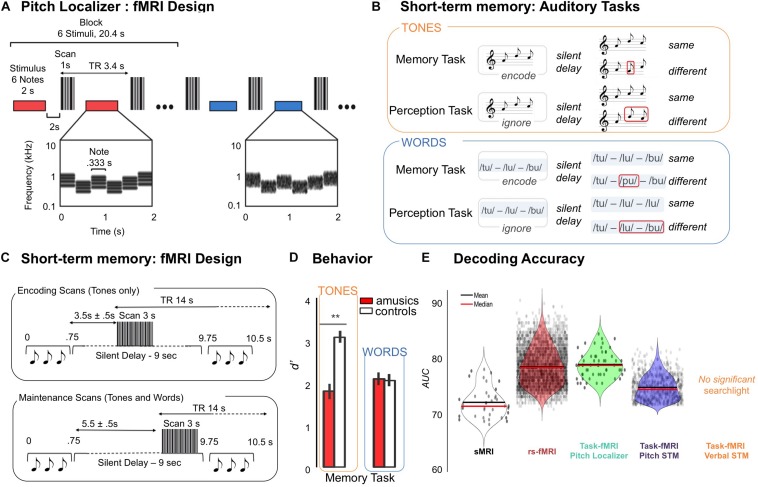
**(A)** Pitch localizer, schematic of the experimental design. fMRI responses were measured to harmonic tones and Gaussian noise spanning the same frequency range. Stimuli (denoted by horizontal bars) were presented in a block design, with six stimuli from the same condition presented successively in each block (red and blue indicate different conditions). Each stimulus (2 s) included several notes that varied in frequency to minimize adaptation. Cochleograms are shown for an example harmonic tone stimulus (red bar) and an example noise stimulus (blue bar). Cochleograms plot time–frequency decompositions, similar to a spectrogram, that summarize the cochlea’s response to sound. After each stimulus, a single scan was collected (vertical, gray bars). Adapted from Norman-Haignere et al. (2016). **(B)** Auditory tasks. Examples of the stimuli used in Memory and Perception Tasks. Memory Task: Participants had to compare sequences (tones or words) presented in pairs. For “same” trials the first sequence was repeated as the second sequence of the pair after a 9000 ms delay. For “different” trials, the second sequence of the pair changed only for one item (in positions 1 to 3, red square). For tonal material, the new item changed the melodic contour. Perception Task: Participants had to compare the two last items (tones or words) of the second sequence regardless of the first sequence. For “same” trials, the two last items of the second sequence were identical. For “different” trials, the two last items of the second sequence were different. Adapted from Albouy et al. (2019). **(C)** Design for the fMRI experiment and timeline of events during one trial. S1 sequence (pitch sequences, words) lasted 750 ms and was followed by a constant 9000 ms silent delay during which occurred 3000 ms of functional data acquisition which was followed by the second sequence (750 ms). Participants had 2000 ms to respond, the next trial occurring 2500 to 3000 ms after the end of S2. A 0 to 500 ms jitter was added at the beginning of the trial to maximize the detection of the BOLD response for the task. As a function of the run, the acquisition of the whole brain volume was realized at two different time periods. Left panel: For Encoding runs (two runs, pitch Material only), acquisition started 3500 to 4000 ms after the end of the S1 sequence. For Maintenance runs (two runs for pitch tasks and two runs for verbal tasks), the volume acquisition occurred just before the second sequence (at the end of the silent delay), the acquisition thus starting from 5500 to 6000 ms after the end of S1. Adapted from Albouy et al. (2019). **(D)** Right Panel: Performance of amusic and control groups (white, Controls; red, Amusics) in terms of dprime, presented as a function of Material (pitch, words), and Group (amusics, controls) for the short-term memory tasks. Error bars indicate SEM. Adapted from Albouy et al. (2019). **(E)** Group classification results for structural (sMRI), resting state functional connectivity (rs-fMRI), and task related fMRI [pitch localizer (PL); pitch memory (PM); verbal memory (VM)]. Results are expressed as area under the receiver-operator-characteristic curve (AUC). AUC uses the distance of a classification output to the decision boundary. Violin plots represent the mean and the median of the AUC in brain regions that were significantly classifying amusics and controls as revealed by searchlight analysis (black dots indicate significant searchlights for each analysis).

During fMRI acquisition, participants performed four tasks: a memory task and a perception task for piano tones, and a memory task and a perception task for monosyllabic words (see Figure 1B). For the tonal tasks both encoding and maintenance were investigated, whereas for the verbal task only maintenance was investigated, so there were two times more trials for the tonal tasks. For all tasks, at each trial, two sequences (of words or tones) were presented sequentially and separated by a silent delay. In the memory task, participants were required to indicate whether the two sequences were the same or different. In the perception task, they were required to ignore the first sequence and indicate whether the last two items of the second sequence were the same or different. The perception task was designed as a control condition: participants listened passively to the same stimuli (i.e., the first sequence) as the one used in the memory task, but without actively encoding the information in memory. All tasks involved two three-sound (words or tones) sequences (S1, S2), separated by a silent maintenance period of 9 s. For both tonal and verbal materials, each sound had a duration of 250 ms, and the three sounds were presented successively with an inter-stimulus-interval of 0 ms.

For the tonal material, 120 different three-tone melodies (that were used as S1 for the 120 tonal trials, 60 for the memory task, 60 for the perception task, see below) were created using eight piano tones differing in pitch height (Cubase software, Steinberg), all belonging to the key of C Major [C3, D3, E3, F3, G3, A3, B3, and C4, material from Albouy et al. (2013a)]. For the verbal material, 60 different sequences (that were used as S1 for the 60 verbal trials, 30 for the memory task, 30 for the perception task, see below) were created using six monosyllabic French words with fixed F0: toux (/tu/- cough), loup (/lu/- wolf), boue (/bu/- dirt), mou (/mu/- soft), goût (/gu/- taste) and pou (/pu/- bug), spoken by a female voice [material from Tillmann et al. (2009)]. F0 of verbal recordings were set constant to 230 Hz (within the range of the piano tones used in the tonal tasks) with STRAIGHT (Kawahara and Irino, 2004), and equalized in loudness using MATLAB software [material adapted from Tillmann et al. (2009)]. The words were selected from a pool of recorded words judged as intelligible by eight native French speakers. For verbal and tonal material, half the S1 sequences contained items repetition (words or tones) in the second and third position of the sequence and the other half did not contain item repetition within the sequence (Figure 1B).

#### Memory Tasks

There were 60 memory trials (S1, silence, S2) for tones and 30 memory trials (S1, silence, S2) for words, each set being equally composed of 50% same and 50% different trials. For different trials, one item of the S2 sequence was different from the S1 sequence (in positions 1 to 3, equally distributed across trials). For melodies, this new item created a contour-violation in the melody. The pitch interval size between the original tone in S1 and the changed tone in S2 was above the PDT of all participants and controlled so that there were 50% of the trials with a medium interval size (of 1.5, 2, and 2.5 tones in equal proportion) and 50% of trials with a large interval size (of 3, 3.5, and 4 tones). For verbal sequences, the changed word was selected from the remaining words that were not presented in the S1 sequence.

#### Perception Tasks

The perception task consisted of 60 trials (S1, silence, S2) for tones and 30 trials (S1, silence, S2) for words (see Figure 1A). Trials were divided into same and different. Importantly, S1 sequences in perception trials were not strictly identical to S1 sequence in memory trials, to avoid exact stimulus repetition, but were similar in terms of melodic contour for the tonal material.

#### Procedure

Amusic and control participants performed the four tasks during fMRI recording. Presentation software (Neurobehavioral Systems, Albany, CA, United States) was used to run the experiment and to record button presses. Stimuli were presented via MRI-compatible insert earphones (*NordicNeuroLab*, in Lyon and *Etymotic Research* in Montreal). The level of sound presentation was set to 70 dB SPL for all participants. The experiment was divided into six runs of about 9 min each: 4 runs with tonal material (2 runs for tonal encoding, 2 runs for tonal maintenance) and 2 runs with verbal material (verbal maintenance). Within a run, memory and perception tasks were presented in blocks of 15 trials each and the task order was counterbalanced across runs and participants. At the beginning of each run, 5 trials of silence served as baseline. Task instructions were presented visually at the beginning and at the middle of each run. During fMRI acquisition, participants were asked to keep their eyes closed. When the task changed, participants heard a salient tone burst, looked at the visual instruction on the screen, and closed their eyes again. The runs were separated by 2–3 min of break. Participants were informed about the material (tones or words) and the order of the to-be-performed tasks before each run.

For each trial within a run, participants indicated their answers by pressing one of two keys of a response device with their right hand after the end of S2. They had 2 s to respond before the next trial, which occurred between to 2.5 s and 3.0 s after the end of S2. In each task, trials were presented in a pseudo-randomized order with the constraint that the same trial type (same or different) could not be repeated more than three times in a row. Before entering the scanner, participants performed 15 practice trials for each task (with simulated scanner noise) with response feedback. No feedback was given during the main experiment.

#### fMRI Design and Acquisition Parameters

A gradient-echo EPI pulse sequence was used to measure whole-brain blood oxygenation level-dependent (BOLD) signal (47 axial slices acquired in ascending sequential order; TR, 14000 ms; volume acquisition, TA = 3000 ms; TE, 30 ms; FA, 90°; 3 mm slice thickness; no gap; matrix size, 80 × 80; FOV 240 mm × 240 mm; voxel size, 3 mm × 3 mm × 3 mm). The long TR (14 s including 3 s of image acquisition, TA) is related to the sparse-sampling paradigm that was used to maximize task-related BOLD response and minimize auditory masking due to MRI scanning noise (Belin et al., 1999). Auditory events were synchronized with fMRI image volume acquisitions at a rate of one image per trial. Within different blocks, we aimed to capture the hemodynamic response associated with two different processes. First, the activity related to the maintenance of the tonal and verbal stimuli was measured with fMRI volumes acquired 5500 to 6000 ms after the end of S1 (Figure 1C, lower panel), thereby decreasing the likelihood of capturing the activity related to the encoding of the S1 stimulus. In two additional runs, we measured the activity related to the encoding of the tonal stimuli (Figure 1C upper panel, with fMRI volumes acquired 3500 to 4000 ms after the end of S1, i.e., at the expected peak of the hemodynamic response for auditory processing of S1). The encoding scans were performed only for the tonal material (2 runs). Note that the maintenance scans were performed for both verbal and tonal materials (2 runs each).

#### Preprocessing

All image preprocessing was performed using SPM12 (Wellcome Trust Centre for Neuroimaging^1^, London, United Kingdom). Before preprocessing, all images were checked for artifacts and automatically aligned so that the origin of the coordinate system was located at the anterior commissure. Preprocessing included the realignment of functional images and the co-registration of functional and anatomical data. We then performed a spatial normalization (voxel size, 3 × 3 × 3) of the T1 and the EPI images to the Montreal Neurological Institute templates provided with SPM12 (MNI T1 template and EPI template respectively). Finally, functional images were spatially smoothed (Gaussian kernel, 5 mm FWHM).

#### fMRI Analyses

This analysis includes fMRI data acquired in two scanner sites (Lyon, Montreal). Multicenter studies can entrain site-dependent effects in fMRI sensitivity, notably regarding activation effect sizes. Friedman and Glover (Friedman and Glover, 2006) have suggested that these confounding effects are mainly linked to different field strength, hardware, and software used in different centers. In the present study, we used similar hardware, software, update version, fMRI sequences, and head coil in the two MRI centers in order to reduce the risk of scanner site effect. Individual contrast maps were first calculated for each participant. A hemodynamic response function (HRF) was chosen to model the BOLD response such that it accounted for the long TR of 14 s (micro time resolution of 80 ms; micro time onset 1; high-pass filter 360-s). At the first level, for each participant, changes in brain regional responses were estimated by a general linear model (GLM) (Friston et al., 1995) and the following memory vs. perception contrast was performed. Contrasts were computed for the combined [encoding and maintenance scans] (for tonal material), and for maintenance scan only for verbal material. These contrasts maps were used in the multivariate analyses.

### Task-fMRI: Pitch Localizer

#### Participants

Note that only a subset of subjects has participated to the task fMRI: pitch localizer (*n* = 12 in each group, all participants were recorded in Lyon). See Supplementary Table S2 for details.

#### Stimuli

Stimuli were composed of either harmonic tone complexes or Gaussian noise (see Figure 1A for a schematic of the design). The tone complexes contained harmonics 3–6 of their fundamental frequency (F0). We did not include the fundamental frequency or second harmonic in the stimulus because they are not needed to produce a robust pitch percept (Houtsma and Smurzynski, 1990) and because their inclusion produces an excitation pattern (the average cochlear response as a function of frequency) that more substantially differs from that of noise due to their wide spacing in the cochlea. Gaussian noise sounds were filtered to span the same frequency range as the harmonic tone complexes. Each stimulus lasted 2 s and included 6 “notes” that were varied in frequency to minimize adaptation (for details, see Norman-Haignere et al., 2013). For each stimulus, the overall frequency range across all notes spanned either a low or high spectral region. We used two frequency ranges so that we could also test for tonotopic organization as a positive control in case amusic participants showed weaker or absent pitch responses. Our analyses focused on characterizing pitch-responsive voxels by contrasting responses to harmonic tones and noise, combining across the two frequency ranges (frequency-selective responses reflecting tonotopy were evident in both groups, as expected). To assess pitch responses, we contrasted responses to harmonic tones and noise, and summed this contrast across both low- and high-frequency ranges to maximize statistical power: [low tones - low noise] + [high tones - high noise]. The mean F0s for the low- and high-frequency harmonic notes were 166 and 666 Hz, respectively (yielding frequency ranges of the harmonics spanning 0.5–1 and 2–4 kHz, respectively). Noise was not used to mask cochlear distortion products because for spectrally ‘resolvable’ harmonics like those tested here, distortion products have little effect on the response of pitch regions (Norman-Haignere and McDermott, 2016).

To focus subjects’ attention on the stimuli, participants performed a rhythm judgment task intended to be similarly difficult for amusics and controls: each stimulus had notes of either equal durations (333 ms) or irregular durations (183–583 ms), and subjects were instructed to indicate whether they heard a regular or irregular rhythm using a button press. Performance on the rhythm task was similar between amusics and controls, with no significant group difference [*t*(20) = 1.42; *p* = 0.17].

#### Procedure

Stimuli were presented in a sparse, blocked design, with 6.2 s stimuli from the same condition presented successively in each block (Figure 1A). After each stimulus, a single scan was collected (Belin et al., 1999). Each participant completed a single run of the experiment, which included five blocks for each of the four conditions and five blocks of silence to provide a baseline with which to compare responses (each block lasted 20.4 s). Condition orders were pseudorandom and counterbalanced across participants: for each participant, a set of condition orders was selected from a large set of randomly generated orders (20,000) such that, on average, each condition was equally likely to occur at each point in the run and each condition was preceded equally often by every other condition in the experiment. Presentation software (Neurobehavioral Systems) was used to present sounds in the scanner and record button responses. Sounds were presented at a fixed level (70 dB SPL) using MRI-compatible earphones (Nordic NeuroLab).

#### Data Acquisition and Preprocessing

The details of the scanning sequence were identical to that used in Norman-Haignere et al. (2013). Briefly, each functional volume (e.g., a single 3D image) comprised 15 slices covering most of the superior temporal cortex and oriented parallel to the superior temporal plane (slices were 4 mm thick with a 2.1 mm × 2.1 mm in-plane resolution). Volumes were acquired every 3.4 s. Each acquisition lasted 1 s and stimuli were presented in the 2.4 s gap of silence between acquisitions (Figure 1B). Functional volumes were motion corrected and aligned to the anatomical volume from each participant. Head motion and voxel SNR were similar between the two groups, with no significant difference in either measure [*t*(20) < 0.2, *p* > 0.8 for both]. The aligned volumes were resampled to the high-density surface mesh computed by FreeSurfer for each individual participant; and these individual-participant meshes were aligned to the mesh of a standardized template brain (the MNI305 FsAverage brain). Note that this mapping to surface was done only for the Pitch localizer to allow a comparison between univariate results reported in the original paper (Norman-Haignere et al., 2016) and multivariate results presented in the current study using analysis using exactly similar data. After alignment, the mesh data were smoothed using a relatively small kernel (3 mm FWHM) and interpolated to a 1.5 × 1.5 mm grid using a flattened representation of the surface mesh. We used a slightly larger smoothing kernel to compute the group-averaged, whole-brain maps described below (5 mm FWHM) to account for the local variability of cortical responses across participants.

#### Regression Analyses

Each voxel was fit with a GLM, with one regressor per stimulus condition. The regressors for each stimulus condition were computed in the standard way, using an estimate of the HRF. This HRF estimate was calculated from the data using a finite-impulse response (FIR) model, rather than assuming a fixed parametric form. To model sources of noise, we included the following nuisance regressors: a linear-trend regressor (to account for signal drift) and the first 10 principal components from voxel responses in white matter (to account for sources of noise with high variance across voxels).

#### Estimating the Hemodynamic Response Function

Each time point in the HRF was modeled with a separate “candlestick” regressor, with a 1 for all scans that occurred a fixed time delay after the onset of a stimulus block (regardless of stimulus type/condition) and a 0 for all other scans. These candlestick regressors were fit to each voxel’s response using ordinary least squares. The weights for each regressor, which collectively provide an estimate of each voxel’s HRF, were then averaged across voxels and participants. We averaged responses across the 10% of voxels in the superior temporal plane (the anatomical region most responsive to sound) of each participant that were best explained by the candlestick regressors (the estimated HRF was robust to the exact number of voxels selected; e.g., selecting the top 50% of voxels yielded similar results). This analysis provided an estimate of the average HRF to a stimulus block in our experiment across all conditions and participants. Regressors for each condition and each participant were computed from this HRF and fit to the voxel responses.

#### Whole-Brain Contrast Maps

We calculated maps showing voxels with a significant response preference for sounds with pitch (harmonic tones > noise). Each voxel’s response time course was fit with the four stimulus regressors and 11 nuisance regressors described above. The weights for the tone and noise regressors were subtracted and then summed across the two frequency ranges (i.e., [low tones – low noise] + [high tones – high noise]). This difference score for each voxel and participant was converted to a z-statistic (using ordinary least-squares equations). These z-maps were then mapped back to the volume using FreeSurfer to perform the searchlight analyses (see below).

### Multivariate Analysis

We were interested in classifying participants as amusic or control according to their structural MRI, resting state connectivity maps and task-related fMRI data. Imaging metrics for task-based fMRI were optimized for univariate analyses: the fMRI designs were defined to generate bold signal associated with the tasks of interest (Pitch localizer, short-term memory for tones and short-term memory for words). For sMRI we focused on whole brain GM and WM volumes for which abnormalities have already been reported with univariate analyses in congenital amusia. Similarly, for resting state, we investigated whole brain connectivity patterns with seeds in bilateral auditory cortices for which abnormal connectivity with the default mode network have been reported. Multivariate analyses were performed using the Decoding Toolbox (Hebart et al., 2014) and LibSVM’s linear support vector machine (SVM) implementation^5^. A linear classifier was chosen as MRI (sMRI, fMRI) data contains many more features than examples, and classification of such data is generally susceptible to over-fitting. One way of alleviating the danger of over-fitting is to choose a simple function (such as a linear function) for classification, where each feature affects the prediction solely via its weight and without interaction with other features (rather than more complex classifiers, such as non-linear SVMs or artificial neural networks, which can let interactions between features and non-linear functions thereof drive the prediction). Linear SVMs are pairwise classifiers; we thus ran analyses on pairs of “conditions” (i.e., amusic group vs. control group). We used motion corrected, normalized, and smoothed data.

All classification analyses were performed using a leave-one-out cross-validation procedure. For example, the classifier was trained on data from 35 of the images and tested on data from the 36^th^ image, repeated 36 times. In all analyses, SVM classification was performed using a searchlight procedure (Kriegeskorte et al., 2006) whereby the classification algorithm considers only voxels from a small sphere of space (radius = 12 mm, see Klein and Zatorre (2015) for a similar procedure). The radius of the searchlight was based on the largest voxel size of our five different datasets (3 × 3 × 3). Twelve mm corresponds to four voxels in the fMRI data, a radius classically used in fMRI literature (see guidelines of the software we used (Hebart et al., 2014) and previous work from our lab (Klein and Zatorre, 2015). We decided to use the similar radius for all analyses in order to be able to compare the classification outputs for the different imaging metrics.

Results are expressed as area under the receiver-operator-characteristic curve (AUC) of category identification, which uses the distance of a classification output to the decision boundary and can provide results about the information content using a graded rather than a binary response (see Hebart et al., 2014). AUC was calculated using an average of the cross-validation folds, and this value was assigned to the center voxel of the sphere. This procedure was repeated using every brain voxel as a searchlight center (∼35,000–45,000 spheres), yielding local accuracy maps for the entire brain. The analysis output was a unique map containing for each voxel the classification AUC.

### Statistical Analysis

To assess whether these classification values were significant, we compared maps of classification accuracy with a null distribution of permutations. Each permutation was constructed by randomly reordering group labels and by repeating the same analysis 1000 times thus providing a null distribution that was used for assessing significance. To correct for multiple comparisons, we used a simple variant of cluster-correction suited for the permutation test (Norman-Haignere et al., 2016). For each set of permuted condition orders, we computed a map of voxelwise significance values using the permutation analysis just described. We then thresholded this uncorrected voxelwise significance map (*p* < 0.05) and recorded the size of the largest contiguous voxel cluster that exceeded this threshold. Using this approach, we built up a null distribution for cluster sizes across the 1000 permutations. To evaluate significance, we counted the fraction of times that the cluster sizes for this null distribution exceeded that for each observed cluster (based on un-permuted orders and the same *p* < 0.05 voxelwise threshold). For significant brain regions, we extracted the decision values (estimated for each cross validation fold separately and indicating the distance of each participant to the separating hyperplane) of the statistical peak (maximum zscore after permutation testing and cluster correction) to estimate Pearson’s correlation with behavioral data.

The behavioral data consisted in participants’ accuracy for the short-term memory task for pitch sequences described above that has been performed by all 36 participants. The pitch short-term memory tasks was used instead of the Montreal Battery of Evaluation of amusia (MBEA; Peretz et al., 2003) to perform correlations with brain classification because: (1) the participants were defined as amusics or controls based on the MBEA scores only and thus, (2) the pitch memory task constituted an independent behavioral metric that (3) shows an overlap in performance between amusics and controls (see Figure 1D). This task thus allowed us to investigate if participants who can potentially be misclassified in brain imaging data as amusics (or controls) show comparable performance in the pitch memory task.

Note that this potential link between classification outcome and behavior was not estimated for the task-fMRI tonal short-term memory, as the correlation would have been performed with behavioral data acquired during the actual fMRI recording. This analysis would thus not be comparable with the correlation analyses performed between the behavioral data (tonal short-term memory task) and the other imaging metrics (sMRI, rs-fMRI, task-fMRI Pitch Localizer) that have been acquired either without or with a different behavioral task.

Finally, is it relevant to note that the tonal short-term memory task is correlated with the MBEA (see Albouy et al., 2019), and the MBEA has been used behaviorally to define group membership. One can argue that a significant correlation might be driven by the group difference. To avoid this possible effect, for significant correlations, a null distribution of *r*-values was generated by permuting 10,000 times the behavioral data within each group. To evaluate significance, we estimated the fraction of times that the *r*-value for this null distribution exceeded that of the un-permuted data. With is approach, if an observed correlation is a consequence of the group membership and nothing more, it should not matter which individual has which score and, we would expect to get the same magnitude of correlation than with the un-permuted (original) data. In contrast, if the probability of obtaining *r*-value for this null distribution that exceeds that of the un-permuted data is below *p* = 0.05 we could reasonably argue that a circularity argument could not account for the findings.

## Results

Whole-brain searchlight analyses (Support Vector Machine, leave-one-out cross-validation procedure, permutation statistics, and cluster-level corrections) were performed on five different datasets consisting in: (A) a set of gray and white matter concentrations maps extracted from T1-MPRAGE volumes (data from Albouy et al., 2019); (B) whole-brain rs-fMRI seed-based connectivity maps with seeds in the right and left auditory cortices (data from Leveque et al., 2016); and (C) three task-fMRI datasets (data from Norman-Haignere et al., 2016; Albouy et al., 2019) consisting of: a pitch localizer (Figure 1A), a short-term memory task for pitch (Figures 1B–D), and a short-term memory task for words (control dataset, Figures 1B–D). Data are from 36 subjects (18 amusics – 18 controls) who participated to the structural imaging (T1-MPRAGE) and Task-fMRI for short-term memory (tones, words). Note that only a subset of these subjects participated in the rs-fMRI (13 in each group, all participants were recorded in Lyon) and task fMRI: pitch localizer (*n* = 12 in each group, all participants were recorded in Lyon).

Figure 1D illustrated the behavioral performance of amusics and controls for these short-term memory tasks, where a Group (amusics, controls) by Material (pitch, words) interaction [*F*(1,34) = 18.42, *p* < 0.001] revealed decreased performance in amusics as compared to controls for pitch memory (*p* < 0.001), but not for verbal memory (*p* = 0.99, Tukey corrected). fMRI designs are depicted in Figures 1A,C.

The classification was successful (see details below, Figure 1E) in right temporal and frontal brain regions and DMN, for all datasets, except for the task-related fMRI acquired during the verbal memory task, as predicted from the literature (see Figure 1E). Note that classification accuracy [estimated as area under the receiver-operator-characteristic curve (AUC)] was not significantly different (McNemar tests-corrected for multiple comparisons performed on AUC values of the significant searchlights; all *p*s > 0.39) between the successful classifiers. Below we describe these results for each dataset.

### Structural MRI

**FIGURE 2:**
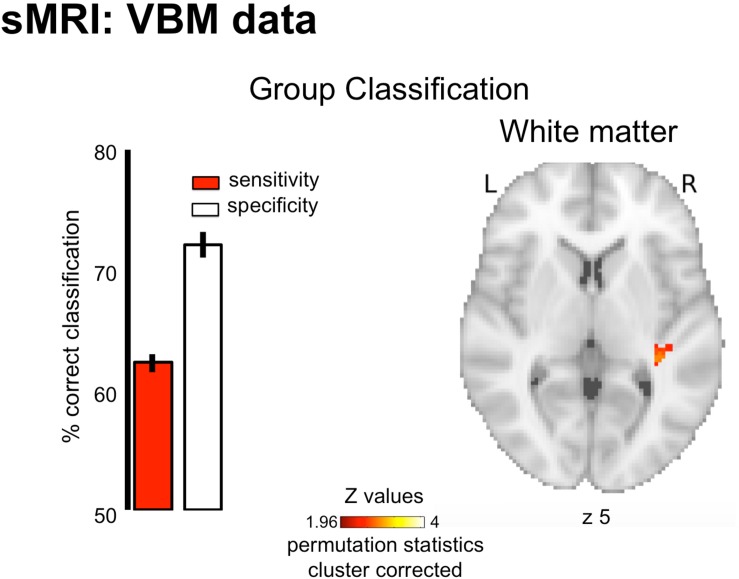
Group classification results for structural data (White Matter). Results are displayed on a single participant T1 in the MNI space provided by SPM12. Bar plots represent sensitivity (red) and specificity (white) of the classifier.

The pattern of white-matter concentration in the right STG (Figure 2, peak at x = 36 y = −32 z = 5; cluster size k = 39; cluster-level corrected *p* = 0.026) discriminated amusics from controls significantly above chance level (AUC 71.69% ± 0.45 SEM of significant searchlights, *p* < 0.05). However, this classifier showed lower sensitivity (62.25 ± 0.79%, percentage of amusics correctly identified as amusics) than specificity (72.07 ± 1.07%, percentage of controls correctly identified as controls), thus questioning its predictive capacity (high rate of false negatives). This was also illustrated by the absence of correlation between the classifier decision values and participant behavioral performance in the short-term memory task for pitch [*r*(34) = −0.09, *p* > 0.05]. Finally, the classification based on gray-matter maps showed a unique cluster in the right Inferior frontal gyrus (permutation testing), but this effect did not survive cluster correction (see Supplementary Figure S1).

### Resting-State fMRI

**FIGURE 3:**
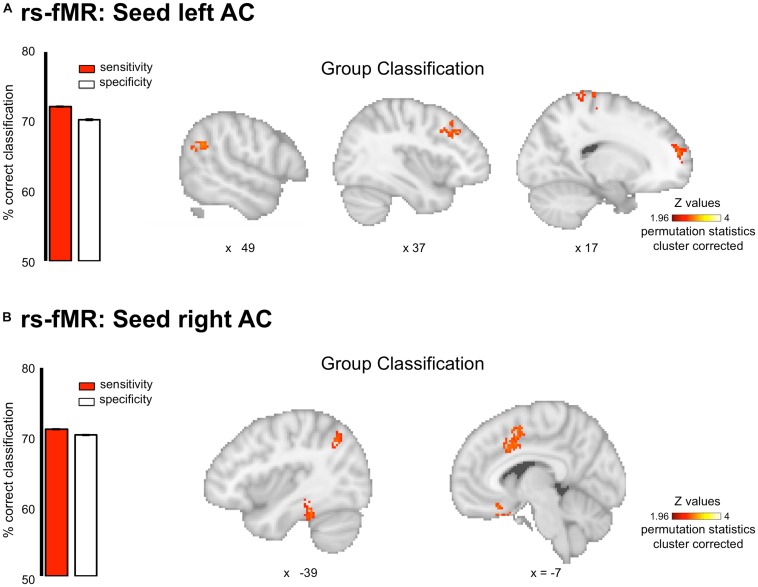
Group classification for the resting state connectivity data. Classification was performed on whole brain connectivity maps with seeds in the left **(A)** and right **(B)** auditory cortices. Results are displayed single participant T1 in the MNI space provided by SPM12. Bar plots represent sensitivity (red) and specificity (white) of the classifier.

For resting-state data, the MVPA analyses revealed that the pattern of connectivity between the auditory cortices and the default mode network allowed classifying amusics vs. controls. Indeed, classification based on connectivity maps for seeds in the right and left Heschl’s gyri (MNI coordinates (x = 45 y = −19 z = 6) and (x = −44 y = −18 z = 5), coordinates from Albouy et al., 2013a) showed high AUC (right seed: 77.93 ± 0.07%, *p* < 0.05; left seed: 78.68 ± 0.09%) in several clusters (see Figure 3 and Table 2 for details) of the Default Mode Network (as revealed by the overlap between the significant clusters of the present study and a mask of the DMN extracted from a coordinate-based meta-analysis^6^ (Acikalin et al., 2017).

**TABLE 2:** Coordinates of regions of significantly above chance level decoding for the rs-fMRI data.

**Seed**	**H**	**Regions**	**x y z mm**	**Cluster size**	***p*-cluster-level corrected**
Right AC	L	Middle cingulate gyrus	−10 10 42	606	*p* = 0.001
		Gyrus rectus^∗^	−4 23 −24	313	*p* = 0.01
		Angular gyrus^∗^	−35 −63 45	432	*p* = 0.003
		Inferior temporal gyrus	−57 −38 −23	528	*p* = 0.001
Left AC	R	Angular gyrus^∗^	57 −50 28	336	*p* = 0.008
		Middle frontal gyrus^∗^	41 26 35	404	*p* = 0.003
		Superior frontal gyrus	17 58 25	259	*p* = 0.02
		Post-central gyrus	17 −36 77	258	*p* = 0.021

*Coordinates are in the MNI space and correspond to the peak of each cluster. R, right; L, left; AC, auditory cortex.^∗^Clusters overlapping with the DMN (see https://identifiers.org/neurovault.collection:1653, Acikalin et al., 2017).*

Interestingly, similar sensitivity and specificity were observed (right seed: sensitivity 71.09 ± 0.18%, specificity 70.33 ± 0.19%; left seed: sensitivity 72.13 ± 0.21%, specificity 70.26 ± 0.20%), confirming the predictive capacity of the classifier. However, classifier decision values were not correlated with participant behavioral performance in the short-term memory task for pitch [all *r*s(20) < 0.29, all *p*s > 0.05].

### Task-fMRI: Short-Term Memory

**FIGURE 4:**
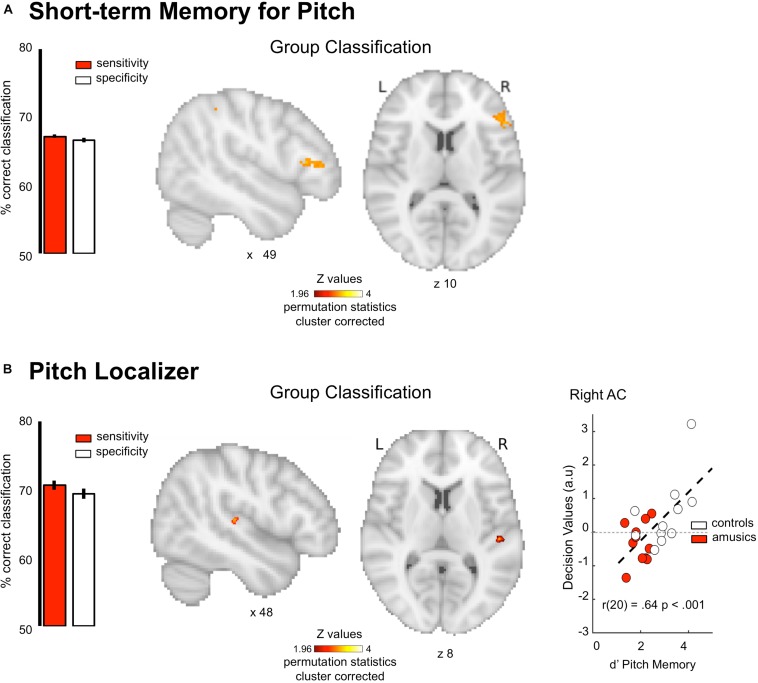
Group classification results for task-related functional imaging. **(A)** Group classification for the tonal short-term memory data. **(B)** Group classification for the pitch localizer data. Scatter plot indicates classification decision values against behavioral performance in a pitch memory task Results are displayed single participant T1 in the MNI space provided by SPM12. Bar plots represent sensitivity (red) and specificity (white) of the classifier.

fMRI responses were measured to pitch memory and pitch perception trials (Figure 1A, see section “Materials and Methods”). The perception task was designed as a control condition: participants listened passively to the same stimuli (i.e., the first sequence) as the one used in the memory task, but without actively encoding the information in memory (Figure 1B). By contrasting memory and perception trials, we aimed to identify the brain networks specifically related to short-term memory processes in each group. The MVPA analyses were thus performed on first-level contrast maps (different of beta weights) for the contrast [memory–perception]. We found that the pattern of functional activity during pitch memory in the right IFG (triangular part, x = 48 y = 34 z = 10, k = 240, cluster-level corrected *p* = 0.001) discriminates amusics from controls significantly above chance level (Figure 4A, AUC 74.45 ± 0.07%, Sensitivity 67.19 ± 0.28%, Specificity 66.62 ± 0.35%). As mentioned in Section “Materials and Methods,” the correlation between behavioral data and classifier outcome was not evaluated for the task-fMRI tonal short-term memory, as the correlation would have been performed with behavioral data acquired during the actual fMRI recording. Finally, as predicted, the classifier was not able to decode the groups on the task-related fMRI data for verbal material (Figure 1E, no significant cluster).

### Task-fMRI: Pitch Localizer

fMRI responses were measured to harmonic tones and Gaussian noise spanning the same frequency range (Figure 1A), while participants performed a non-pitch related task focusing on rhythmic features. We then calculated maps showing voxels with a significant response preference for sounds with pitch. The MVPA analyses were done on these first-level z-maps for the contrast [harmonic tones – Gaussian noise]. The pattern of functional activity in the right Heschl’s gyrus (x = 51 y = −23 z = 7, k = 69 cluster-level corrected *p* = 0.001, Figure 4B) discriminates amusics from controls significantly above chance level (AUC 78.67 ± 0.32%, Sensitivity 70.65 ± 0.72%, Specificity 69.37 ± 0.77%). Finally, and more importantly, the classifier decision values were positively correlated with participants’ performance in tonal short-term memory [*r*(20) = 0.64, *p* < 0.001; Figure 4B, right panel]. After generating the null distribution of *r*-values (10k permutations of behavior values within each group), the probability of obtaining *r*-values for this null distribution that exceeds that of the un-permuted data was *p* = 0.01. We could thus reasonably argue that this effect is not driven by group membership (see section “Materials and Methods”). The correlation was significant only for task-based fMRI unlike any of the other imaging metrics reported above: *r*-values were significantly higher than for structural and resting state data (all *p*s < 0.05).

## Discussion

In the present study, we showed that structural MRI, resting-state connectivity, and pitch-related fMRI data (pattern of BOLD activation) were able to discriminate amusic individuals from typical control participants. Decoding was observed in meaningful brain networks as the results reproduce previous univariate results [sMRI (new dataset), rs-fMRI (reused form Leveque et al., 2016) and task-fMRI short-term memory (reused from Albouy et al., 2019)]. The only difference concerns the task fMRI – Pitch localizer (Norman-Haignere et al., 2016) that highlight a group difference in the right auditory cortex during pitch perception. The implications of these findings are discussed below.

For structural MRI, classification analysis highlighted the right STG, a region where decreased white-matter concentration in amusics as compared to controls have been reported (Albouy et al., 2013a). For rs-fMRI, classification revealed several clusters of the DMN, such as medial prefrontal or bilateral inferior parietal regions. This is in line with Leveque et al. (2016), who have interpreted this effect as a marker of incomplete maturation of the auditory networks in congenital amusia.

For task-based fMRI, classification performed during pitch memory revealed that pattern of activity in the right IFG discriminates amusics from controls. This result is in line with previous neuroimaging studies in congenital amusia reporting decreased or abnormal activity in the right IFG during pitch perception and memory (Hyde et al., 2011; Albouy et al., 2013a, 2015, 2019; Peretz, 2016; Tillmann et al., 2016). Interestingly, the classifier was not able to decode the groups on the task-related fMRI data for verbal material, indicating that the classifier outcome is specific to the engagement of neural circuits dedicated to pitch processing (impaired in congenital amusia), but not others. This result also confirms that the relative accuracy of the multivariate classifier (cross-validated) is unlikely to reflect over-fitting. Moreover this observed specificity for pitch-related tasks (disorder related) allows ruling out other confounds such as differences in head motion or attentional differences between the groups.

Finally, the pattern of functional activity in the right Heschl’s gyrus (Pitch localizer dataset) also discriminated amusics from controls. This group difference in the auditory cortex contrasts with a prior fMRI study using the same dataset, where we found that the overall anatomical distribution and selectivity of pitch-responsive voxels was similar between amusics’ and controls (Norman-Haignere et al., 2016). Our new results demonstrate that there are subtle but reliable differences in the pattern of pitch selectivity between amusics and controls in primary auditory cortex, revealing the utility of classification-based approaches compared with standard univariate and region-of-interest (ROI) analyses. Our results are also consistent with prior reports showing abnormal electrophysiological responses in amusics’ auditory cortices using Magnetoencephalography (Albouy et al., 2013a, 2015). Because of fMRI’s superior spatial precision our results reveal a candidate anatomical locus in auditory cortex for pitch-specific deficits in amusia.

Interestingly, successful classifiers (see Figure 1E) were all showing AUC around 70–80%. This level of discrimination power is line with a recent study (Serrallach et al., 2016) showing a classification accuracy of around 70% in MEG and sMRI and psychometric behavioral data using linear regression methods to find specific biomarkers for ADHD, attention deficit disorder (ADD) and dyslexia. It is relevant to note that the authors were able to reach a sensitivity of around 90% when combining all these data in one classification analysis. It would be thus very interesting for future work to estimate the power of such multimodal classification in identifying congenital amusia. Finally, the successful classifiers exhibited similar specificity and sensitivity except for the classification performed on structural data. Indeed decoding observed in the right STG showed lower sensitivity than specificity (see section “Results” or below) suggesting that structural changes can be considered as a less sensitive biomarker for the identification of congenital amusia than the functional changes in the right AC and IFG task-related activity and in the AC resting-state functional connectivity.

When relating the classifier decision values to a behavioral metric, we showed that task based fMRI classification decision values were positively predicting participants’ performance in tonal short-term memory, unlike any of the other imaging data (sMRI, rs-fMRI). The correlation values were significantly different form sMRI and rs-fMRI, thus suggesting that task-related imaging data can be used as a more powerful diagnostic tool than sMRI and rs-fMRI to identify developmental disorders, as it allows defining fine-grained patterns of brain activity that predict behavioral performance and thus yield relevant information about symptom severity.

This study shows the power of task-related fMRI data to identify and predict behavioral performance in congenital amusia. However this study does not allow concluding for the generalizability of this approach. Further work is thus needed to confirm the advantage of task-related fMRI over structural MRI and rs-fMRI to predict symptom severity in other developmental disorders. Moreover, it is relevant to note that the sample size is relatively small for an imaging-based classification analysis (see Varoquaux, 2018). However, our results performed at the whole-brain level highlighted specific brain regions and networks that have previously been reported as abnormal/malfunctioning in congenital amusia, rather than inconsistent or unusual regions that would be more typical of false-positive responses for example.

Overall, our findings show that task-based imaging classifications identify key dysfunctional brain regions and circuits that allow to (1) improve our understanding of the biological basis of neurodevelopmental and learning disorders and (2) predict symptom severity. We propose that such approach might have a beneficial and generalizable impact on diagnosis of developmental and learning disorders, such as dyslexia (Jaffe-Dax et al., 2017) where similar deficits in the ability to perceive and memorize rapidly changing acoustic information have been reported.

## Data Availability Statement

The raw data supporting the conclusions of this manuscript will be made available by the authors, without undue reservation, to any qualified researcher.

## Ethics Statement

The study was approved by French and Canadian local ethics committees on Human Research and all participants gave written informed consent.

## Author Contributions

PA, AC, IP, BT, and RZ: conceptualization. PA, YL, and SN-H: methodology and fMRI pre-processing. PA: data recording, fMRI analysis, writing – original draft, and visualization. AC, BT, IP, and RZ: resources, supervision, and project administration. PA, AC, SN-H, YL, IP, and RZ: writing – review and editing.

## Conflict of Interest

The authors declare that the research was conducted in the absence of any commercial or financial relationships that could be construed as a potential conflict of interest.

## Footnotes

1 http://www.fil.ion.ucl.ac.uk/spm/

2 http://imaging.mrc-cbu.cam.ac.uk/imaging/DataDiagnostics

3 http://dbm.neuro.uni-jena.de/vbm/

4 http://brainvisa.info/

5 http://www.csie.ntu.edu.tw/~cjlin/libsvm/

6 https://identifiers.org/neurovault.collection:1653

## References

[B1] Acikalin M. Y. Gorgolewski K. J. Poldrack R. A. (2017). A coordinate-based meta-analysis of overlaps in regional specialization and functional connectivity across subjective value and default mode networks. *Front. Neurosci.* 11:1. 10.3389/fnins.2017.00001 28154520 PMC5243799

[B2] Albouy P. Mattout J. Bouet R. Maby E. Sanchez G. Aguera P. E. (2013a). Impaired pitch perception and memory in congenital amusia: the deficit starts in the auditory cortex. *Brain* 136 1639–1661. 10.1093/brain/awt082 23616587

[B3] Albouy P. Schulze K. Caclin A. Tillmann B. (2013b). Does tonality boost short-term memory in congenital amusia? *Brain Res.* 1537 224–232. 10.1016/j.brainres.2013.09.003 24041778

[B4] Albouy P. Mattout J. Sanchez G. Tillmann B. Caclin A. (2015). Altered retrieval of melodic information in congenital amusia: insights from dynamic causal modeling of MEG data. *Front. Hum. Neurosci.* 9:20. 10.3389/fnhum.2015.00020 25698955 PMC4316716

[B5] Albouy P. Peretz I. Bermudez P. Zatorre R. J. Tillmann B. Caclin A. (2019). Specialized neural dynamics for verbal and tonal memory: fMRI evidence in congenital amusia. *Hum. Brain Mapp.* 40 855–867. 10.1002/hbm.24416 30381866 PMC6916746

[B6] Arbabshirani M. R. Plis S. Sui J. Calhoun V. D. (2017). Single subject prediction of brain disorders in neuroimaging: promises and pitfalls. *Neuroimage* 145 137–165. 10.1016/j.neuroimage.2016.02.079 27012503 PMC5031516

[B7] Ashburner J. Friston K. J. (2005). Unified segmentation. *Neuroimage* 26 839–851. 10.1016/j.neuroimage.2005.02.018 15955494

[B8] Belin P. Zatorre R. J. Hoge R. Evans A. C. Pike B. (1999). Event-related fMRI of the auditory cortex. *Neuroimage* 10 417–429. 10.1006/nimg.1999.0480 10493900

[B9] Bray S. Chang C. Hoeft F. (2009). Applications of multivariate pattern classification analyses in developmental neuroimaging of healthy and clinical populations. *Front. Hum. Neurosci.* 3:32. 10.3389/neuro.09.032.2009 19893761 PMC2773173

[B10] Brett M. Anton J. L. Valabregue R. Poline J. B. (2002). Region of interest analysis using the marsbar toolbox for SPM 99. *Neuroimage* 16:497.

[B11] Bruin W. Denys D. Van Wingen G. (2018). Diagnostic neuroimaging markers of obsessive-compulsive disorder: initial evidence from structural and functional MRI studies. *Prog. Neuropsychopharmacol. Biol. Psychiatry* 91 49–59. 10.1016/j.pnpbp.2018.08.005 30107192

[B12] Caclin A. Tillmann B. (2018). Musical and verbal short-term memory: insights from neurodevelopmental and neurological disorders. *Ann. N. Y. Acad. Sci.* 10.1111/nyas.13733 [Epub ahead of print]. 29744897

[B13] Fauvel B. Groussard M. Chetelat G. Fouquet M. Landeau B. Eustache F. (2014). Morphological brain plasticity induced by musical expertise is accompanied by modulation of functional connectivity at rest. *Neuroimage* 90 179–188. 10.1016/j.neuroimage.2013.12.065 24418502

[B14] Friedman L. Glover G. H. (2006). Report on a multicenter fMRI quality assurance protocol. *J. Magn. Reson. Imaging* 23 827–839. 10.1002/jmri.20583 16649196

[B15] Friston K. Holmes A. Worsley K. J. Poline J. B. Frith C. D. Frackowiak R. S. J. (1995). Statistical parametric maps in functional imaging: a general linear approach. *Hum. Brain Mapp.* 2 189–210. 10.1002/hbm.460020402

[B16] Greicius M. D. Krasnow B. Reiss A. L. Menon V. (2003). Functional connectivity in the resting brain: a network analysis of the default mode hypothesis. *Proc. Natl. Acad. Sci. U.S.A.* 100 253–258. 10.1073/pnas.0135058100 12506194 PMC140943

[B17] Hebart M. N. Gorgen K. Haynes J. D. (2014). The decoding toolbox (TDT): a versatile software package for multivariate analyses of functional imaging data. *Front. Neuroinform.* 8:88. 10.3389/fninf.2014.00088 25610393 PMC4285115

[B18] Houtsma A. J. M. Smurzynski J. (1990). Pitch identification and discrimination forcomplex tones with many harmonics. *J. Acoust. Soc. Am.* 87 304–310. 10.1121/1.399297

[B19] Hyde K. L. Lerch J. P. Zatorre R. J. Griffiths T. D. Evans A. C. Peretz I. (2007). Cortical thickness in congenital amusia: when less is better than more. *J. Neurosci.* 27 13028–13032. 10.1523/jneurosci.3039-07.2007 18032676 PMC6673307

[B20] Hyde K. L. Zatorre R. J. Griffiths T. D. Lerch J. P. Peretz I. (2006). Morphometry of the amusic brain: a two-site study. *Brain* 129 2562–2570. 10.1093/brain/awl204 16931534

[B21] Hyde K. L. Zatorre R. J. Peretz I. (2011). Functional MRI evidence of an abnormal neural network for pitch processing in congenital amusia. *Cereb. Cortex* 21 292–299. 10.1093/cercor/bhq094 20494966

[B22] Jaffe-Dax S. Frenkel O. Ahissar M. (2017). Dyslexics’ faster decay of implicit memory for sounds and words is manifested in their shorter neural adaptation. *Elife* 6:e20557. 10.7554/eLife.20557 28115055 PMC5279949

[B23] Kawahara H. Irino T. (2004). “Underlying principles of a high-quality speech manipulation system STRAIGHT and its application to speech segregation,” in *Speech Separation by Humans and Machines*, ed. Divenyi P. L. (Alphen aan den Rijn: Kluwer Academic), 167–180. 10.1007/0-387-22794-6_11

[B24] Klein M. E. Zatorre R. J. (2015). Representations of invariant musical categories are decodable by pattern analysis of locally distributed bold responses in superior temporal and intraparietal sulci. *Cereb. Cortex* 25 1947–1957. 10.1093/cercor/bhu003 24488957 PMC4459292

[B25] Kriegeskorte N. Goebel R. Bandettini P. (2006). Information-based functional brain mapping. *Proc. Natl. Acad. Sci. U.S.A.* 103 3863–3868. 10.1073/pnas.0600244103 16537458 PMC1383651

[B26] Leveque Y. Fauvel B. Groussard M. Caclin A. Albouy P. Platel H. (2016). Altered intrinsic connectivity of the auditory cortex in congenital amusia. *J. Neurophysiol.* 116 88–97. 10.1152/jn.00663.2015 27009161 PMC4961744

[B27] Loui P. Alsop D. Schlaug G. (2009). Tone deafness: a new disconnection syndrome? *J. Neurosci.* 29 10215–10220. 10.1523/JNEUROSCI.1701-09.2009 19692596 PMC2747525

[B28] Norman-Haignere S. Kanwisher N. Mcdermott J. H. (2013). Cortical pitch regions in humans respond primarily to resolved harmonics and are located in specific tonotopic regions of anterior auditory cortex. *J. Neurosci.* 33 19451–19469. 10.1523/JNEUROSCI.2880-13.2013 24336712 PMC3916670

[B29] Norman-Haignere S. McDermott J. H. (2016). Distortion products in auditory fMRI research: measurements and solutions. *Neuroimage* 129 401–413. 10.1016/j.neuroimage.2016.01.050 26827809 PMC4803580

[B30] Norman-Haignere S. V. Albouy P. Caclin A. Mcdermott J. H. Kanwisher N. G. Tillmann B. (2016). Pitch-responsive cortical regions in congenital amusia. *J. Neurosci.* 36 2986–2994. 10.1523/JNEUROSCI.2705-15.2016 26961952 PMC6601753

[B31] Peretz I. (2016). Neurobiology of congenital amusia. *Trends. Cogn. Sci.* 20 857–867. 10.1016/j.tics.2016.09.002 27692992

[B32] Peretz I. Champod A. S. Hyde K. (2003). Varieties of musical disorders. *Ann. N. Y. Acad. Sci.* 999 58–75. 10.1196/annals.1284.006 14681118

[B33] Raichle M. E. Macleod A. M. Snyder A. Z. Powers W. J. Gusnard D. A. Shulman G. L. (2001). A default mode of brain function. *Proc. Natl. Acad. Sci. U.S.A.* 98 676–682. 11209064 10.1073/pnas.98.2.676 PMC14647

[B34] Serrallach B. Gross C. Bernhofs V. Engelmann D. Benner J. Gundert N. (2016). Neural biomarkers for dyslexia. *Front. Neurosci.* 10:324. 10.3389/fnins.2016.00324 27471442 PMC4945653

[B35] Shenas S. K. Halici U. Cicek M. (2014). A comparative analysis of functional connectivity data in resting and task-related conditions of the brain for disease signature of OCD. *Conf. Proc. IEEE Eng. Med. Biol. Soc.* 2014 978–981. 10.1109/EMBC.2014.6943756 25570124

[B36] Tillmann B. Leveque Y. Fornoni L. Albouy P. Caclin A. (2016). Impaired short-term memory for pitch in congenital amusia. *Brain Res.* 1640 251–263. 10.1016/j.brainres.2015.10.035 26505915

[B37] Tillmann B. Schulze K. Foxton J. M. (2009). Congenital amusia: a short-term memory deficit for non-verbal, but not verbal sounds. *Brain Cogn.* 71 259–264. 10.1016/j.bandc.2009.08.003 19762140

[B38] Uddin L. Q. Dajani D. R. Voorhies W. Bednarz H. Kana R. K. (2017). Progress and roadblocks in the search for brain-based biomarkers of autism and attention-deficit/hyperactivity disorder. *Transl. Psychiatry* 7:e1218. 10.1038/tp.2017.164 28892073 PMC5611731

[B39] Varoquaux G. (2018). Cross-validation failure: small sample sizes lead to large error bars. *Neuroimage* 180 68–77. 10.1016/j.neuroimage.2017.06.061 28655633

[B40] Williamson V. J. Stewart L. (2010). Memory for pitch in congenital amusia: beyond a fine-grained pitch discrimination problem. *Memory* 18 657–669. 10.1080/09658211.2010.501339 20706954

