# Supplementary material for: Decoding Task-Related Functional Brain Imaging Data to Identify Developmental Disorders: The Case of Congenital Amusia
Source: Front Neurosci. 2019 Oct 30;13:1165. doi: 10.3389/fnins.2019.01165 (PMC6831619; doi:10.3389/fnins.2019.01165)
Supplement: Supplementary file 1 [file Table_1.DOCX]

**Supplementary Material**

**Decoding task-related functional brain imaging data to identify developmental disorders: the case of congenital amusia**

Philippe Albouy, Anne Caclin, Sam V. Norman-Haignere, Yohana Lévêque^,^, Isabelle Peretz, Barbara Tillmann, and Robert J. Zatorre

*
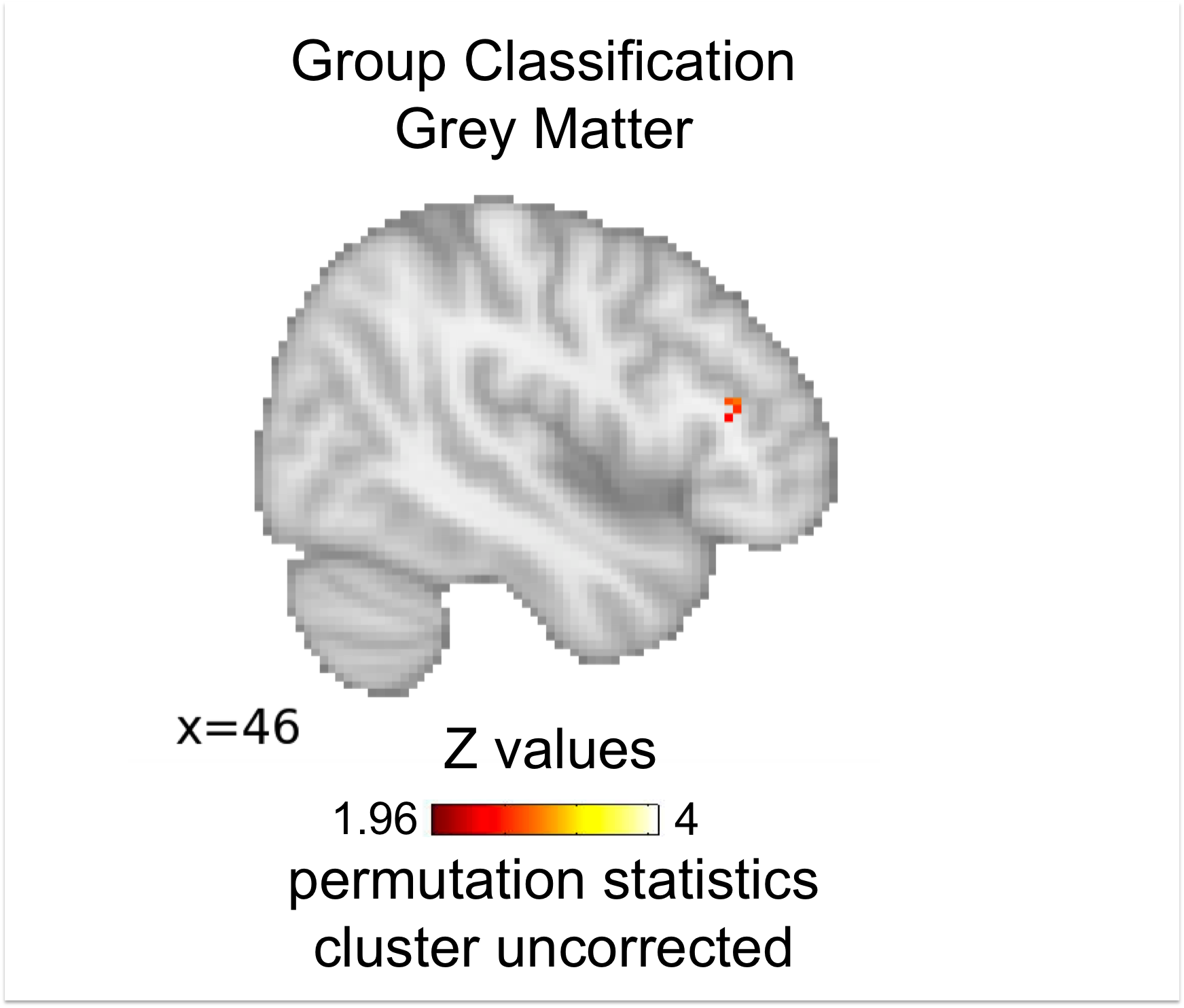
*

Supplementary Figure 1: Group classification results for Grey matter data. Results are displayed on a single participant T1 in the MNI space provided by SPM12.

| Demographic Characteristics | Amusics (n=13) | Controls (n=13) | t-Test |
| --- | --- | --- | --- |
| Age in years | 37.08 (13.6) | 36.92 (10.99) | *p = .58 (NS)* |
| Gender | 7F, 6M | 7F, 6M | *N/A* |
| Education in years | 13.46 (2.14) | 13.76 (3.1) | *p = .22 (NS)* |
| Musical education in years | 0.76 (1.53) | 0.15 (.55) | *p = .18 (NS)* |
| MBEA [14] | 21.4 (1.24) | 26.88 (1.53) | *p < .0001* |
|  |  |  |  |

*Supplementary Table S1: Demographic characteristics of amusics and controls who participated in resting state protocol. Results on the Montreal Battery of Evaluation of Amusia (MBEA) are expressed as number of correct responses (average over the six sub-tests of the battery, maximum score = 30). Data are reported as a function of group and groups are compared with t-tests. ‘‘NS’’ refers to a non-significant difference (p>.05) and standard deviations are in parentheses.*

| Demographic Characteristics | Amusics (n=12) | Controls (n=12) | t-Test |
| --- | --- | --- | --- |
| Age in years | 36.33 (13.9) | 36.34 (12.69) | *p = .99 (NS)* |
| Gender | 7F, 5M | 6F, 6M | *N/A* |
| Education in years | 13.16 (1.94) | 13.08 (2.8) | *p = .93 (NS)* |
| Musical education in years | 0.41 (.90) | 0.01 | *p = .12 (NS)* |
| MBEA [14] | 21.34 (1.28) | 26.58 (1.65) | *p < .0001* |
|  |  |  |  |

*Supplementary Table S2: Demographic characteristics of amusics and controls who participated in Pitch Localizer. Results on the Montreal Battery of Evaluation of Amusia (MBEA) are expressed as number of correct responses (average over the six sub-tests of the battery, maximum score = 30). Data are reported as a function of group and groups are compared with t-tests. ‘‘NS’’ refers to a non-significant difference (p>.05) and standard deviations are in parentheses.*
